# Prevalence of Overweight/Obesity Associated With Anemia Among Female Medical Students at Umm Al-Qura University in Makkah, Saudi Arabia: A Cross-Sectional Study

**DOI:** 10.7759/cureus.57081

**Published:** 2024-03-27

**Authors:** Munerah Hamed, Amal Zaghloul, Saeed H Halawani, Bushra A Fatani, Bashair Alshareef, Aisha Almalki, Esraa Alsharif, Qamar A ALhothaly, Salma Alhadhrami, Hanan M Abd Elmoneim

**Affiliations:** 1 Department of Pathology, Faculty of Medicine, Umm Al-Qura University, Makkah, SAU; 2 Department of Clinical Pathology (Hematology), Faculty of Medicine, Ain Shams University, Cairo, EGY; 3 Department of Hematology and Immunology, Faculty of Medicine, Umm Al-Qura University, Makkah, SAU; 4 Department of Medicine and Surgery, Faculty of Medicine, Umm Al-Qura University, Makkah, SAU

**Keywords:** medical students, overweight, obesity, iron deficiency anemia, anemia

## Abstract

Introduction

The obesity epidemic has been linked to a wide range of health and nutritional problems, including anemia, which is often caused by impaired iron metabolism. The World Health Organization considers anemia and obesity to be global health issues among adolescent girls and women experiencing menstruation. This study aims to examine the association between iron deficiency anemia and obesity/overweight among female medical students.

Methodology

This cross-sectional descriptive study conducted as an online self-administered questionnaire. Furthermore, blood samples were collected from 206 students to evaluate the complete blood count, iron and lipid profile.

Results

The convenience sampling technique was used and a total of 206 students were enrolled in the study. The average body mass index (BMI) was 22.51 ± 3.25, with 83.5% (n = 172) falling within the normal weight range, 12.6% (n = 26) as overweight, and 3.9% (n = 8) as obese. Anemia was present in 16.0% (n = 33) of the participants overall. Higher prevalence of anemia was observed among overweight participants with 10 out of 26 (38.5%) subjects compared to obese with two out of eight (25.0%) and normal weight 21 out of 172 (12.2%); this difference was highly significant (*P* = 0.005). Individuals with anemia exhibited a significant association with those experiencing a diet full of unhealthy fats and carbohydrates (*P *= 0.05) and a diet containing all essential nutrients (*P* = 0.01). There is no statistically significant correlation between anemia prevalence and participants' response to the presence of signs of anemia, physical activity or other dietary habits. Obese participants had a significantly higher mean value of triglycerides (129.5 ± 20.5) compared to normal weight and overweight participants (74.5 ± 12.02 and 51.2 ± 15.04), respectively (*P* = 0.001).

Conclusion

A dependable assembly exists between obesity and overweight in cases of iron deficient anemia. The prevalence of iron deficiency anemia was substantially higher among overweight/obese females, highlighting that overweight/obesity signifies both quantitative and qualitative malnutrition. A high BMI was associated with elevated triglycerides, typically considered indicators of obesity. This association may suggest compromised iron homeostasis.

## Introduction

Obesity and overweight are unhealthy accumulations of excess body fat that can have negative health consequences and higher mortality rates globally [1,2]. One complication of obesity and overweight is anemia, which is a reduction in the overall quantity of red blood cells or levels of hemoglobin, or a combination of both [3,4]. Obesity and overweight can cause low-grade systemic inflammation and increased hepcidin secretion, reducing iron absorption and leading to iron sequestration within various cells, including macrophages, hepatocytes, and enterocytes, contributing to anemia. Furthermore, poor micronutrient intake and high-calorie diet consumption in overweight and obese people can lead to anemia [5].

The Kingdom of Saudi Arabia has undergone significant changes in food consumption and dietary habits, gradually becoming increasingly "Westernized". Such a "nutritious transformation" has been blamed for the recent increase in overweight and obesity rates among the Saudi population [6]. A study that was conducted in 2016-2017 at Princess Nourah Bint Abdulrahman University in Riyadh revealed that the prevalence of overweight/obesity among females in general was (27.1%) and (24.0%), respectively, while in health colleges (College of Medicine, Nursing, Medical Sciences and Pharmacy), the prevalence of overweight and obesity in females was (29.5%) and (38.6%), respectively [7]. Furthermore, another research conducted at King Abdulaziz University, Kingdom of Saudi Arabia, demonstrated that anemia among medical female university students was at a rate of 33.1% [8].

Considering the health implications associated with anemia, the surge in the prevalence of anemia among females, and the global increase in obesity, emphasize the importance of examining the prevalence of obesity and overweight conditions and the association between these conditions with hemoglobin levels and dietary habits. Some studies show that people who are overweight or obese have fewer symptoms of anemia than those who are normal weight [9]. Another study found that anemia is associated with an increased risk of long-term complications of cardiovascular events and death, particularly in obese patients. High iron reserves in the body have been linked to an increased risk of coronary heart disease in several epidemiological studies, with hyperlipidemia being the most critical risk factor [10,11].

A study of young (18-25 years old) general female students at King Abdulaziz University in Saudi Arabia found that the prevalence of iron deficiency anemia was 33.1%. Among the group of students with anemia, 6.9% were classified as overweight, while 4.3% were identified as obese [8]. Another study from Iraq's Al-Kindy College of Medicine/University of Baghdad found that 71.2% of overweight and obese females and 28.8% of overweight and obese males are anemic [4]. Moreover, previous research has shown that pediatric obesity increases the risk of developing multiple childhood and adult comorbidities, such as anemia [2]. Furthermore, other studies reported that overweight and obesity are major risk factors for iron deficiency anemia in adolescents [5,12].

There have been few studies on the relationship between anemia and obesity in Saudi Arabia, and fewer studies have been conducted in Makkah. Furthermore, previous studies on this topic could not clarify the relationship between obesity and anemia. This study forms a crucial aspect of the current investigation, aiming to offer insights for subsequent studies and interventions.

Thus, the present study aimed to determine the association between iron deficiency anemia and obesity/overweight among female medical students. In addition, it seeks to evaluate the occurrence rates of overweight, obesity, and anemia, along with their correlations with dietary patterns, physical activity levels, and overall body fat percentages among female medical students enrolled in Umm Al-Qura University in Makkah.

## Materials and methods

Study design

This study was a cross-sectional descriptive study using an online self-administered questionnaire conducted between July 2021 and August 2022 in the College of Medicine (female section) at Umm Al-Qura University in Makkah, Saudi Arabia.

Sample size and inclusion criteria

The targeted sample size was calculated to be a minimum of 153 using the OpenEpi version 3.0 (https://www.openepi.com/SampleSize/SSPropor.htm) with a confidence level determined as 95.0%. The convenience sampling technique was used and a total of 206 students were enrolled in the study. The inclusion criteria to participate in this study were all adult female medical students of the Faculty of Medicine who agreed to participate (from first to the intern year) and aged 18 years or above. Medical students who had bleeding disorders, chronic diseases such as diabetes or hypertension, menstrual disorders, hematological diseases, were underweight, pregnant or declined to participate in the study were excluded.

Data collection

Blood samples were collected from a pool of 206 students, who also completed a self-administered online questionnaire, by sharing the survey link through social media. The survey included a brief overview of the research's objective and inquiries related to various sociodemographic and physical variables, such as age, weight, height, dietary preferences, consumption of fast food, physical activity, menstrual status, health and psychological factors and finally, lifestyle. Venous blood samples were collected (5 ml) using ethylenediaminetetraacetic acid (EDTA) tubes for hemoglobin assessments. These samples were subsequently transferred into plain test tubes, followed by centrifugation at 4,500 rounds per minute (rpm) for 10 minutes to isolate serum. The samples were then preserved at a temperature of -20°C to facilitate subsequent analysis of iron levels.

Furthermore, in this research context anemia was defined as a hemoglobin concentration below 11.9 g/dL in accordance with the classification for anemia in non-pregnant adult women as established by the World Health Organization (WHO) in 2011 [13]. The study encompassed the evaluation of several variables. The complete blood count (CBC), which includes hemoglobin, red blood cells (RBC), white blood cells (WBC), mean corpuscular volume (MCV), mean corpuscular hemoglobin (MCH), red cell distribution width (RDW) and platelets, were categorized as dependent variables. The iron profile, including serum iron, serum ferritin, total iron binding capacity, and transferrin saturation, were also classified as dependent variables. Furthermore, the lipid profile, including total cholesterol, triglycerides, low-density lipoprotein (LDL), and high-density lipoprotein (HDL), were established as independent variables within the study.

Statistical analysis

Data were analyzed using Statistical Package for Social Science (SPSS) program version 20 (IBM Corp., Armonk, NY, USA). Quantitative data were described as mean ± SD for the normally distributed data. The median, minimum and maximum were used for the data that were not normally distributed. The comparison between groups was performed using the Student's t-test, the Mann-Whitney U test, the Kruskal-Wallis test and the one-way ANOVA test. The chi-square and Fisher exact tests were used to compare qualitative data. P ≤ 0.05 was considered statistically significant.

Ethical consideration

The ethical approval was obtained from the Biomedical Research Ethics Committee at Umm Al-Qura University (certification No. HAPO-02-K-012-2021-04-674). The study was carried out according to the Helsinki update and informed consent was obtained from all subjects involved in the study.

## Results

Table 1 shows that the mean age of participants was 20.76 ± 1.8 years, 25.7% were in the second academic year and 1.9% were married. The majority of the participants have their menstrual cycle for ≤ seven days (84.5%) and 68.0% change their sanitary pads > three times/day during their menstrual cycle. Only 6.8% reported a familial background of obesity. The average BMI was 22.51 ± 3.25, with 83.5% falling within the normal weight range, 12.6% categorized as overweight, and 3.9% classified as obese. In total, the study encompassed 206 female students aged between 18 and 25 years, who were ranked into the following categories based on BMI: those with a BMI below 18.5 kg/m², signifying underweight, which were excluded; individuals falling within the BMI of 18.6 to 24.9 kg/m² were classified as normal weight, constituting 83.5%; participants with a BMI ranging from 25 to 29.9 kg/m² were categorized as overweight, making up 12.6%; and those with a BMI equal to or exceeding 30 kg/m² were designated as individuals with obesity, comprising 3.9%. The mean age among individuals in the normal-weight category was 20.72 ± 1.83 years, whereas in the overweight and obesity group, the mean age was 20.75 ± 1.7 and 21.63 ± 1.59, respectively. Upon assessing BMI, the normal-weight group exhibited a mean BMI of 21.43 ± 1.79 kg/m², while the overweight and obese group demonstrated a mean BMI of 26.97 ± 1.39 and 32.71 ± 2.71, respectively.

**Table 1:** Correlation between anemia and distribution of studied participants according to their demographic data, academic level, menstrual data and family history of obesity Data were expressed as mean +/- standard error of mean. Significance between “anemia” and “no anemia” individuals was made using unpaired Student's t test for parametric parameters and chi-square test for non-parametric parameters.

Variable	No. (%)	Anemia	No Anemia	P-value
		No. (%)	No. (%)	
Age in years	20.76 ± 1.8	20.67 ± 1.35	20.77 ± 1.85	0.1
Academic year				0.1
1st year	27 (13.1)	2 (6.1)	25 (14.5)	
2nd year	53 (25.7)	9 (27.3)	44 (25.4)	
3rd year	20 (9.7)	6 (18.2)	14 (8.1)	
4th year	29 (14.1)	2 (6.1)	27 (15.6)	
5th year	48 (23.3)	10 (30.3)	38 (22)	
6th year	22 (10.7)	2 (6.1)	20 (11.6)	
Intern	7 (3.4)	2 (6.1)	5 (2.9)	
Marital status				0.4
Married	4 (1.9)	0 (0)	4 (2.3)	
Single	202 (98.1)	33 (100)	169 (97.7)	
How many days is your menstrual cycle?				0.3
≤7 days	174 (84.5)	29 (87.9)	145(83.8)	
>7 days	32 (15.5)	4 (12.1)	28 (16.2)	
How many times a day you change your sanitary pads?				0.3
≤3 times	66 (32)	12 (36.4)	54 (31.2)	
>3 times	140 (68)	21 (63.6)	119 (68.8)	
Do you have a family history of obesity?				0.1
No	192 (93.2)	29 (87.9)	163 (94.2)	
Yes	14 (6.8)	4 (12.1)	10 (5.8)	

**Figure 1:**
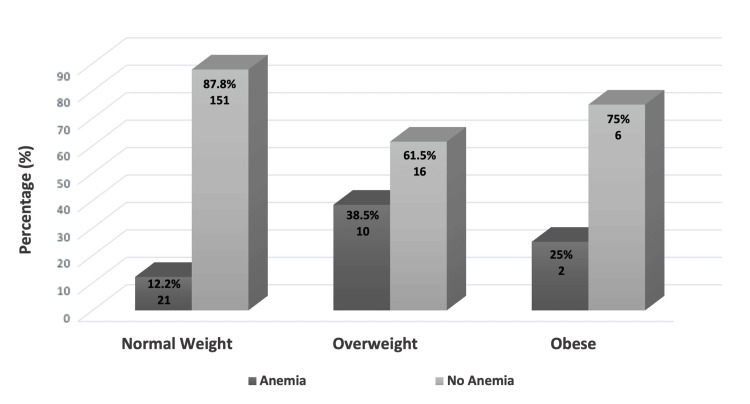
Relationship between BMI categories and the prevalence of anemia (total number of studied participants = 206). “Anemia” represents participants with hemoglobin < 11.9 g/dL, whereas “no anemia” represents participants with hemoglobin ≥ 11.9 g/dL.

The overall prevalence of anemia was 16.0% in the total number of participants. Higher prevalence of anemia among overweight participants (38.5%) compared to those with obesity (25.0%) and normal weight (12.2%); this difference was statistically significant (P = 0.005) (Figure 1). In addition, we assessed the association between the prevalence of anemia and participants' age, academic level, marital status, menstrual cycle number of days, changing of sanitary pads and family history of obesity. Our analysis indicated no statistically significant relationship between anemia and those categories (P = 0.1, P = 0.1, P = 0.4, P = 0.3, P = 0.3 and P = 0.1, respectively) (Table 1).

Table 2 summarizes the correlation between anemia prevalence and participants' response regarding certain factors such as health, lifestyles and physical activities. There is no statistically significant correlation between anemia prevalence and participants' response regarding the presence of signs of anemia and physical activity, other dietary habits or feeling unwilling to go to university or study (P = 0.2). It demonstrates that 50.5% of the participants had fatigue or tiredness, 46.1% suffered from dizziness or headache and 30.6% suffered from poor concentration or academic achievement. Of them, 10.2% suffer from influenza or the common cold continuously, and 58.3% reported that their distress affects their healthy eating and daily lifestyle. Most of them (69.4%) reported that their feeling of distress has a role in changing their physical health, such as feeling tired, exhausted and headache. The majority (84.0%) thought that there is a relationship between the deterioration of mental health and anemia. More than half (51.0%) regularly exercise four times a week and 28.2% follow a diet recently. Moreover, most of the participants (91.7%) thought that there is a relation between an unhealthy diet and anemia. However, 53.4% reported consuming a diet full of unhealthy fats and carbohydrates, almost one-third (33.5%) commit to eating the three main meals breakfast, lunch and dinner, while 42.2% reported having a diet containing all essential nutrients. The majority (70.9%) sometimes feel upset or unwilling to attend university or study. On the other hand, individuals with anemia exhibited a significant association with those consuming a diet full of unhealthy fats and carbohydrates (P = 0.05) and a diet containing all essential nutrients (P = 0.01) (Table 2).

**Table 2:** Correlation between anemia prevalence and participants' response to the presence of signs of anemia and physical activity, dietary habits and feeling unwilling to go to university or study Significance between "anemia” and “no anemia” individuals was made using unpaired Student's t test for parametric parameters and chi-square test for non-parametric parameters.

Variable	No. (%)	Anemia	No Anemia	P-value
		No. (%)	No. (%)	
Have you had fatigue or tiredness?				0.1
No	102 (49.5)	13 (39.4)	89 (51.4)	
Yes	104 (50.5)	20 (60.6)	84 (48.6)	
Do you suffer from dizziness or headache?				0.8
No	111 (53.9)	17 (51.5)	94 (54.3)	
Yes	95 (46.1)	16 (48.5)	79 (45.7)	
Do you suffer from poor concentration or poor academic achievement?				0.3
No	143 (69.4)	20 (60.6)	123 (71.1)	
Yes	63 (30.6)	13 (39.4)	50 (28.9)	
Do you suffer from influenza or the common cold continuously?				0.2
No	185 (89.8)	32 (97)	153 (88.4)	
Yes	21 (10.2)	1 (3)	20 (11.6)	
Is your feeling of distress affecting your healthy eating and daily lifestyle?				0.8
No	86 (41.7)	13 (39.4)	73 (42.2)	
Yes	120 (58.3)	20 (60.6)	100 (57.8)	
Does your feeling of distress have a role in changing your physical health, such as feeling tired, exhausted and headache?				0.89
No	63 (30.6)	9 (27.3)	54 (31.2)	
Yes	143 (69.4)	24 (72.7)	119 (68.8)	
Do you think there is a relationship between the deterioration of mental health and anemia?				0.6
No	33 (16)	4 (12.1)	29 (16.8)	
Yes	173 (84)	29 (87.9)	144 (83.2)	
Did you do any kind of exercise regularly, four times a week?				0.7
No	101 (49)	17 (51.5)	90 (48.6)	
Yes	105 (51)	16 (48.5)	95 (51.4)	
Do you follow a diet recently?				0.09
No	148 (71.8)	20 (60.6)	128 (74)	
Yes	58 (28.2)	13 (39.4)	45 (26)	
Do you think there is a relationship between unhealthy diet and anemia?				0.3
No	17 (8.3)	4 (12.1)	13 (7.5)	
Yes	189 (91.7)	29 (87.9)	160 (92.5)	
Is your diet full of unhealthy fats and carbohydrates?				0.05
No	96 (46.6)	20 (60.6)	76 (43.9)	
Yes	110 (53.4)	13 (39.4)	97 (56.1)	
Do you commit to eating the three main meals breakfast, lunch and dinner?				0.6
No	137 (66.5)	21 (63.6)	116 (67.1)	
Yes	69 (33.5)	12 (36.4)	57 (32.9)	
Does your diet contain all essential nutrients?				0.01
No	119 (57.8)	13 (39.4)	106 (61.3)	
Yes	87 (42.2)	20 (60.6)	67 (38.7)	
Do you feel upset or unwilling to go to university or study?				0.2
Always	19 (9.2)	1 (3)	18 (10.4)	
Rarely	41 (19.9)	9 (27.3)	32 (18.5)	
Sometimes	146 (70.9)	23 (69.7)	123 (71.1)	

Table 3 shows that obese participants had a significantly higher mean value of triglycerides (129.5 ± 20.5) compared to normal weight and overweight participants (74.5 ± 12.02 and 51.2 ± 15.04), respectively (P = 0.001). A non-significant difference was found between studied BMI categories and mean values of hemoglobin (P = 0.92), total cholesterol (P = 0.25), HDL (P = 0.79), LDL (P = 0.65), iron (P = 0.23) or ferritin (P = 0.82).

**Table 3:** Relationship between body mass index (BMI) categories and laboratory analysis Data were expressed as mean +/- standard error of mean. Significance between groups of normal weight, overweight and obese individuals was made using one-way ANOVA for parametric parameters and Kruskal-Wallis test for non-parametric parameters (ferritin).

	BMI category			F-value	P-value
	Normal weight	Overweight	Obese		
						Hemoglobin g/dl	12.57 ± 1.44	12.65 ± 0.84	12.34 ± 2.23	2	0.925
Total cholesterol mg/dl	168.5 ± 31.82	151.47 ± 26.94	189.5 ± 21.92	1.62	0.256
HDL mg/dl	67 ± 4.24	62.4 ± 18.32	56 ± 8.48	0.23	0.797
LDL mg/dl	86.5 ± 33.23	94.08 ± 17.34	107.5 ± 34.64	0.45	0.653
Triglycerides mg/dl	74.5 ± 12.02	51.2 ± 15.04	129.5 ± 20.5	19.95	0.001
Iron ug/dl	72.92 ± 20.69	76.37 ± 33.89	33 ± 1.21	1.57	0.235
Ferritin* ng/ml	27.64 ± 20.97	24.73 ± 11.91	19.55 ± 7.7	0.2*	0.82
MCV fl	83.60 ± 7.35	82.34 ± 5.00	77.81 ± 11.79	2.7	0.19
MCH fl	28.05 ± 2.94	27.43 ± 1.67	27.43 ± 2.79	0.41	0.18

## Discussion

Because overweight and obese people may be more susceptible to anemia, it is critical to do routine tests and provide ongoing care for this demographic. The overall prevalence of anemia in the total number of our study participants was 16.0%, consistent with findings from other studies where anemia prevalence ranged from 13.7% to 18.1% [14,15]. However, in another study, the prevalence of anemia among the total student sample was higher at 33.1% including underweight participants [8]. Discrepancies in anemia prevalence between our study and others may be attributed to several factors, including differences in study populations, methodologies, sample sizes, geographical locations, or time periods. Additionally, variations in study design, data collection methods, or diagnostic criteria for anemia can contribute to differences in reported prevalence rates, as different studies may utilize distinct cutoff values for hemoglobin levels or diagnostic criteria.

Anemia may be prevalent in obese adolescents in their early adolescence, and this should be recognized by clinicians treating or monitoring their health [16]. The current study describes the relationship between anemia status in participants of healthy overweight and obese young women among female medical students at Umm Al-Qura University. It is common to identify abnormal iron status measures in overweight and obese people, indicating either an iron overload or deficiency. When iron requirements rise in adolescence and in morbid obesity in maturity, iron insufficiency poses a unique therapeutic challenge [12]. Because iron metabolism plays such an important part in metabolic illnesses, in-depth studies of the underlying mechanism are important for the prevention, diagnosis, treatment, and prognosis of obesity and its comorbidities and can give medical practitioners new clinical suggestions [17].

In this study, a higher prevalence of anemia among overweight participants (38.5%) compared to those who were obese (25.0%) and those with normal weight (12.2%); this difference was found highly significant (P = 0.005). The prevalence of anemia in our study in relation to BMI is consistent with numerous other studies that revealed mounting proof that iron levels and anemia are linked to obesity. Young female students frequently suffer from anemia; this finding could be related to abrupt changes in lifestyle, such as eating habits and insufficient exercise [3,8,18-20]. The theory linking iron deficiency and obesity states that obese individuals experience low-grade systemic inflammation, which raises hepcidin expression. Hepcidin then reduces iron absorption and downregulates iron levels in plasma by breaking down ferroportin in intestinal enterocytes [2,3,21]. Obesity modifies some of the molecular pathways involved in iron metabolism, lowering its bioavailability, when taking into account the complicated influence of obesity on iron homeostasis [22]. According to recent research, iron levels in the body are linked to both diabetes and obesity, and iron may even exacerbate these conditions [2,17,23].

In contrast, some studies demonstrated no association between anemia and obesity that found that individuals with normal BMI are more likely to be anemic than others and those with high BMI have a lower prevalence of anemia such as the research conducted by ELMoslemany et al. They reported that females with normal BMI had a higher prevalence of anemia (30.0%), whereas it was 20.0% in females with increased BMI. In addition, the Hanafi et al. study group stated that anemia was more prevalent among normal BMI females (45.7%) [24].

Age, gender, pathologic causes, physiological status, nutritional factors, socioeconomic circumstances, and other factors play significant roles in the prevalence of anemia [11]. However, in our study, a non-significant relationship was observed between anemia prevalence and participants' demographic data, including academic level, menstrual data, and family history of obesity. This lack of significance may be attributed to the predominantly young, single participants in our study who have not yet experienced obstetric events. Previous research has demonstrated a significant correlation between anemia and factors such as bleeding during pregnancy, higher parity, and obstetric history [6,7,14]. Consequently, the absence of significant findings in our study could be attributed to the unique characteristics of our sample, which largely comprises individuals in an age group that has not undergone obstetric experiences.

In the present study, obese participants had a significantly higher mean value of triglycerides compared to normal weight and overweight participants, this could be explained by an unhealthy lifestyle. The results of the data analysis demonstrated that obesity markedly raised the levels of several metabolic markers, including triglycerides [8,10,25]. Increased triglyceride levels may cause inflammation and oxidative stress in the non-sickle cell anemia population, hence, modulating vascular function and lipolysis remnants of triglyceride are cytotoxic to macrophages [26]. A previous study revealed that obese participants exhibited increased triglyceride hydrolysis and higher pro-inflammatory adipokine release. Meanwhile, normal-weight participants' triglycerides had higher oleic acid contents, potentially contributing to the postprandial anti-inflammatory effects. Therefore, an intricate relationship exists between adipokines, inflammatory markers, and postprandial lipid metabolism in the setting of teenage obesity [27].

Adolescents with dyslipidemia have lower serum iron levels than others without the condition. The mechanism underpinning obesity and iron deficiency may have also explained the lower serum iron levels in adolescents with dyslipidemia, since they were typically overweight and obese. Furthermore, the iron hypothesis states that hepcidin's down-regulation of ferroportin causes excessive iron sequestration inside macrophages to stimulate oxidative processes and the uptake of lipids [21]. In patients with high cardiovascular risk, who are overweight or obese, anemia is linked to a higher risk of long-term unfavorable cardiovascular events and mortality [28].

Many patients who have iron deficiency anemia are asymptomatic; others have vague symptoms of fatigue, headache, irritability, or depression. Our study revealed a non-significant relationship between anemia and participants who suffered from dizziness or headache (P = 0.8). These findings indicate that in resource-limited settings, where routine assessment and identification of anemia may be challenging, a thorough clinical evaluation of individuals should be employed to detect anemia or prioritize them for further biochemical assessment. It is important to note that this approach would be more effective in identifying severe cases of anemia rather than mild forms of the condition [15].

Initially, our study's power might be diminished by the comparatively small sample size. Furthermore, several variables not addressed in this study, such as nutritional consumption, sexual development, and intestinal parasite infections, could potentially cause anemia. Also, the generalizability of the study findings might be limited due to the convenient sampling method used for participant recruitment. Despite these limitations, our findings highlight the current understanding of anemia's epidemiology and relationship to fat and overweight/obese. Lastly, these results may allow for additional research on anemia, a national health issue that requires immediate attention.

## Conclusions

A plausible link exists between iron deficiency anemia and overweight/obesity. The risk of iron deficiency anemia was substantially more prevalent in the overweight/obese females demonstrating that overweight/obesity represents both a quantitative and qualitative form of malnutrition. Herein, a high BMI linked to elevated triglycerides, which are indications of obesity. It may also be a sign of compromised iron homeostasis.

## References

[1] Flegal KM, Carroll MD, Kit BK, Ogden CL (2012). Prevalence of obesity and trends in the distribution of body mass index among US adults, 1999-2010. JAMA.

[2] González-Domínguez Á, Visiedo-García FM, Domínguez-Riscart J, González-Domínguez R, Mateos RM, Lechuga-Sancho AM (2020). Iron metabolism in obesity and metabolic syndrome. Int J Mol Sci.

[3] Guglielmi V, D'Adamo M, Bellia A (2015). Iron status in obesity: an independent association with metabolic parameters and effect of weight loss. Nutr Metab Cardiovasc Dis.

[4] Al-Attar Z, Jasim S, Hashim I, Badai S (2020). Prevalence of anemia types among overweight and obese patients attending the Obesity Research and Therapy Unit at Al-Kindy College of Medicine. Int Med J.

[5] De Andrade Cairo RC, Rodrigues Silva L, Carneiro Bustani N, Ferreira Marques CD (2014). Iron deficiency anemia in adolescents; a literature review. Nutr Hosp.

[6] Elsoadaa SS (2013). Overweight and obesity among Saudi female population. J Am Sci.

[7] Naguib R, Tawfik MM, Alsubaiei SA (2020). Study of bodyweight and eating attitude among female university members in the Kingdom of Saudi Arabia: a comparison between different methods of weight assessment. J Family Med Prim Care.

[8] Al-Zahrani M, Balgoon M, Alkhattabi N (2019). Association between the iron status and body mass index of female students in the Faculty of Science of King Abdulaziz University. J Biochem Tech.

[9] Qin Y, Melse-Boonstra A, Pan X (2013). Anemia in relation to body mass index and waist circumference among Chinese women. Nutr J.

[10] Avila F, Echeverría G, Pérez D (2015). Serum ferritin is associated with metabolic syndrome and red meat consumption. Oxid Med Cell Longev.

[11] Guglielmi V, D'Adamo M, Bellia A, Ciotto RT, Federici M, Lauro D, Sbraccia P (2015). Iron status in obesity: an independent association with metabolic parameters and effect of weight loss. Nutr Metab Cardiovasc Dis.

[12] Aigner E, Feldman A, Datz C (2014). Obesity as an emerging risk factor for iron deficiency. Nutrients.

[13] (2011). Haemoglobin concentrations for the diagnosis of anaemia and assessment of severity. https://www.who.int/publications/i/item/WHO-NMH-NHD-MNM-11.1.

[14] Al Sabbah H (2020). Prevalence of overweight/obesity, anaemia and their associations among female university students in Dubai, United Arab Emirates: a cross-sectional study. J Nutr Sci.

[15] Mehdad S, Benaich S, Hamdouchi AE (2022). Association between overweight and anemia in Moroccan adolescents: a cross-sectional study. Pan Afr Med J.

[16] Jeong J, Cho Y, Cho IY, Ahn J (2022). Association between obesity and anemia in a nationally representative sample of South Korean adolescents: a cross-sectional study. Healthcare (Basel).

[17] Qiu F, Wu L, Yang G (2022). The role of iron metabolism in chronic diseases related to obesity. Mol Med.

[18] Huang YF, Tok TS, Lu CL, Ko HC, Chen MY, Chen SC (2015). Relationship between being overweight and iron deficiency in adolescents. Pediatr Neonatol.

[19] Wang T, Gao Q, Yao Y (2023). Causal relationship between obesity and iron deficiency anemia: a two-sample Mendelian randomization study. Front Public Health.

[20] Aguree S, Owora A, Hawkins M, Reddy MB (2023). Iron deficiency and iron deficiency anemia in women with and without obesity: NHANES 2001-2006. Nutrients.

[21] Zhu Y, He B, Xiao Y, Chen Y (2019). Iron metabolism and its association with dyslipidemia risk in children and adolescents: a cross-sectional study. Lipids Health Dis.

[22] Citelli M, Fonte-Faria T, Nascimento-Silva V (2015). Obesity promotes alterations in iron recycling. Nutrients.

[23] Horinouchi Y, Ikeda Y, Tamaki T (2019). [Body iron accumulation in obesity, diabetes and its complications, and the possibility of therapeutic application by iron regulation]. Nihon Yakurigaku Zasshi.

[24] El-Moslemany AG, Elmoslemany AG, Elbbandrawy AM, Elhosary EA, Gabr AA (2019). Relation between body mass index and iron deficiency anemia in adolescent females. Current Science International.

[25] Cheng HL, Bryant CE, Rooney KB, Steinbeck KS, Griffin HJ, Petocz P, O'Connor HT (2013). Iron, hepcidin and inflammatory status of young healthy overweight and obese women in Australia. PLoS One.

[26] Wang L, Gill R, Pedersen TL, Higgins LJ, Newman JW, Rutledge JC (2009). Triglyceride-rich lipoprotein lipolysis releases neutral and oxidized FFAs that induce endothelial cell inflammation. J Lipid Res.

[27] García-Rodríguez S, Espinosa-Cabello JM, García-González A, González-Jiménez E, Aguilar-Cordero MJ, Castellano JM, Perona JS (2024). Interplay of postprandial triglyceride-rich lipoprotein composition and adipokines in obese adolescents. Int J Mol Sci.

[28] Winther SA, Finer N, Sharma AM, Torp-Pedersen C, Andersson C (2014). Association of anemia with the risk of cardiovascular adverse events in overweight/obese patients. Int J Obes (Lond).

